# Embolization with or without portal vein stenting for bleeding ectopic jejunal varices in hepatopetal portal collaterals due to extrahepatic portal vein occlusion or stenosis after hepatobiliary and pancreatic surgery

**DOI:** 10.1007/s11604-024-01616-1

**Published:** 2024-06-27

**Authors:** Satoyuki Ogawa, Akira Yamamoto, Atsushi Jogo, Ken Kageyama, Etsuji Sohgawa, Go Ohira, Kenjiro Kimura, Shogo Tanaka, Ryosuke Amano, Shigekazu Takemura, Takeaki Ishizawa, Yukio Miki

**Affiliations:** 1 Department of Diagnostic and Interventional Radiology, Graduate School of Medicine, Osaka Metropolitan University, 1-4-3 Asahi-Machi, Abeno-Ku, Osaka, 545-8585 Japan; 2 Department of Diagnostic Radiology, Osaka Saiseikai Nakatsu Hospital, 2-10-39 Shibata, Kita-Ku, Osaka, 530-0012 Japan; 3 Department of Hepato-Biliary-Pancreatic Surgery Graduate School of Medicine, Osaka Metropolitan University, Osaka, Japan

**Keywords:** Ectopic varix, Hepatopetal portal collateral, Varix embolization, Jejunal varix

## Abstract

**Purpose:**

To evaluate the efficacy and safety of embolization with or without portal vein stenting for bleeding ectopic jejunal varices in the hepatopetal portal collateral due to extrahepatic portal vein occlusion or stenosis after hepatobiliary and pancreatic surgery.

**Materials and methods:**

This study included consecutive patients who underwent embolization for bleeding ectopic jejunal varices in the hepatopetal collateral due to extrahepatic portal vein occlusion or stenosis after hepatobiliary and pancreatic surgery between September 2012 and December 2020. The safety, technical and clinical success rates (no re-bleeding within 1 month) and re-bleeding-free survival after the first therapy and overall survival were assessed.

**Results:**

Fourteen sessions in 11 patients were included. Four patients (7 sessions) underwent variceal embolization only, and the remaining seven patients (7 sessions) underwent portal vein stenting and variceal embolization. Technical success was achieved in all 14 sessions (100%). Clinical success was achieved in 13 of 14 sessions (92.9%). No treatment-related serious complications including liver failure were observed. One-year and 2-year re-bleeding-free survival rate after the first endovascular therapy in all 11 patients was 90.9 and 60.6%, respectively. Two patients who experienced re-bleeding had repeat embolization treatment. There was no significant difference in re-bleeding-free survival after endovascular therapy between the combination with stenting and embolization group and the embolization-only group (*p* = 0.13).

**Conclusion:**

Embolization with or without portal vein stenting of bleeding ectopic jejunal varices in the hepatopetal portal collateral due to extrahepatic portal vein occlusion or stenosis after hepatobiliary and pancreatic surgery can be considered a safe, effective, and repeatable therapy for long-term hemostasis of uncontrollable bleeding.

## Introduction

**Fig. 1:**
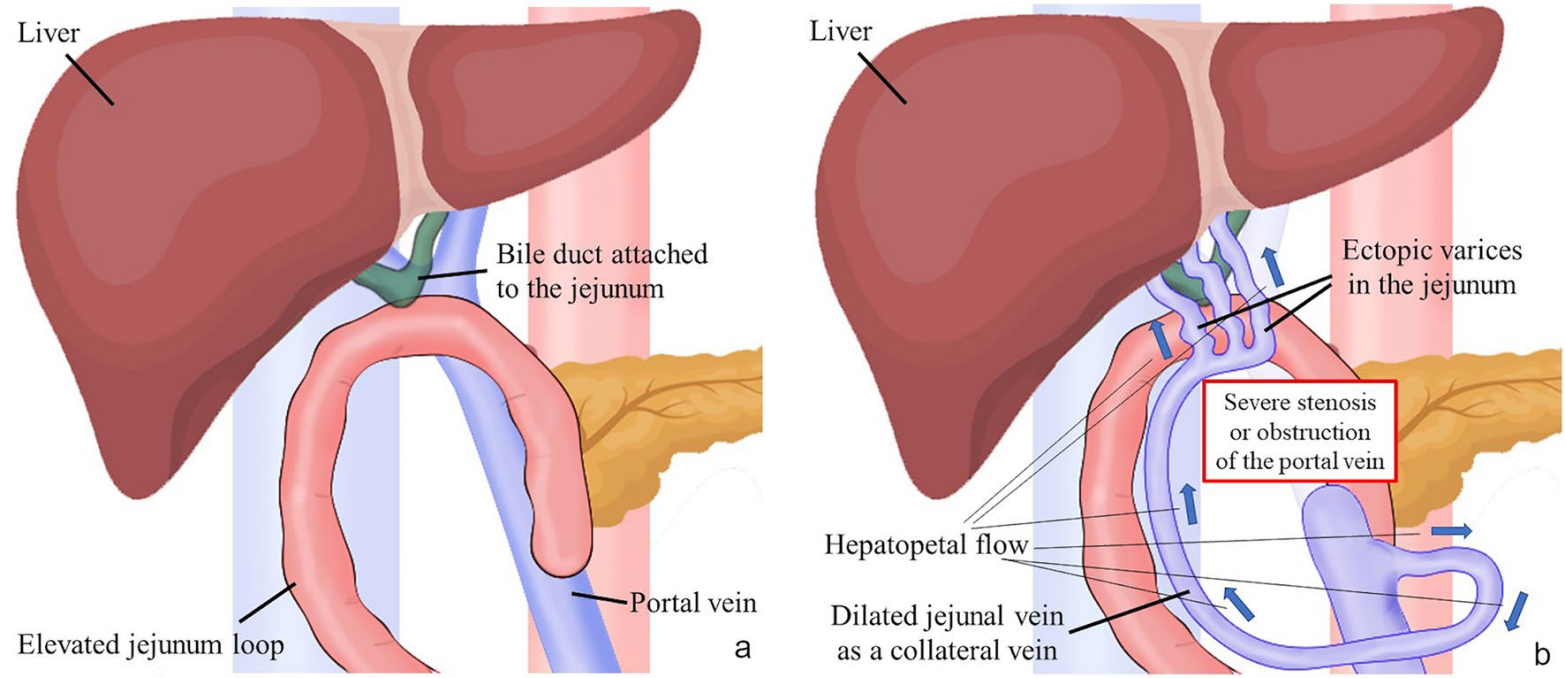
Mechanism of formation of ectopic jejunal varices in hepatopetal portal collaterals after hepatobiliary and pancreatic surgery. **a** After pancreaticoduodenectomy, the bile duct and remaining pancreas are attached to the jejunum. Portal vein flow into the liver without varix formation. **b** After severe stenosis or obstruction of the main branches of the portal vein. Once the portal vein undergoes severe stenosis or occlusion, collateral veins develop, usually as a dilated jejunal vein. Portal blood forms ectopic varices in the elevated jejunal vein near the anastomosis between the bile duct and elevated jejunum. Hepatopetal portal vein flows into the intrahepatic portal vein via the dilated peribiliary plexus

Ectopic varices are defined as dilated portal collateral veins in a location other than the stomach or esophagus, such as the duodenum, jejunum, rectum, stoma or anastomotic site [1–3]. Hepatopetal ectopic varices may occur in the elevated jejunum loop due to stenosis or obstruction of the extrahepatic portal vein after surgery for hepatobiliary and pancreatic diseases (Fig. 1a, b). Ectopic variceal bleeding are rare, accounting for between 1 and 5% of all variceal bleeding [4]. Varices of the jejunal loop are classified as anastomotic varices, and the frequency of anastomotic varices is even lower, representing 5.8% of ectopic varices [2]. Because of the rarity of this disease and the difficulty of performing endoscopic diagnosis and treatment, ectopic variceal bleeding can prove fatal.

The treatment options for ectopic varices are conservative, endoscopic, endovascular, and surgical. Conservative treatment is often inadequate and endoscopic treatment could represent the first-line therapy [4], although reaching afferent jejunal sites can be difficult after surgery due to the anatomical structures. Endovascular treatment may be a good option in terms of reduced invasiveness. Several studies have reported the efficacy of stenting for portal vein occlusion or stenosis and variceal embolization as endovascular treatments [5–12]. However, in some cases of portal vein obstruction, portal vein stenting itself is not feasible and the only endovascular treatment option is variceal embolization. Moreover, malignant stenosis of the portal vein shows a significantly shorter stent patency time than benign stenosis due to tumor recurrence and occlusion by thrombus [13], and variceal embolization with or without portal vein stenting may be effective in preventing bleeding due to short-term variceal recurrence after stent occlusion.

To our knowledge, few case reports have described variceal embolization. The purpose of this study was to evaluate the efficacy and safety of embolization for ectopic jejunal varices in hepatopetal portal collaterals due to extrahepatic portal vein occlusion or stenosis.

## Materials and methods

This retrospective study was approved by the ethics committee at our institution and the requirement for informed consent was waived. We performed a keyword search for the terms “ectopic jejunal varices” and “embolization” in endovascular treatment reports from the radiology imaging system of our institution for the period from September 2012 through December 2020. Consecutive patients who matched these search terms and who underwent embolization for bleeding from ectopic jejunal varices in hepatopetal collaterals due to extrahepatic portal vein occlusion or stenosis were included in the study. These patients showed progressive anemia, ectopic jejunal varices were noted on contrast-enhanced computed tomography (CECT), and endoscopy and hemorrhage scintigraphy were also performed in some cases, leading to a final diagnosis of bleeding from ectopic jejunal varices. Endovascular treatment was indicated for these patients when daily blood transfusions failed to improve anemia and conservative treatment failed to achieve spontaneous hemostasis. The following data were obtained for each patient: primary disease, surgical procedure, cause of portal stricture or occlusion, time to bleeding after surgery, period to retreatment in cases of second or subsequent treatment, follow-up period after first treatment, access route, treatment method, technical success, clinical success, post-treatment complications, re-bleeding-free survival after therapy, stent patency period and overall survival. Causes of portal vein stenosis and obstruction were used to classify patients into a benign stenosis group and a tumor recurrence group. Benign stenosis was caused by inflammation due to chronic pancreatitis and pancreatic fistula. Technical success was defined as complete embolization of the variceal route that was inferred to represent the source of bleeding. Clinical success was defined as no re-bleeding within 1 month as reported by Shim et al. [9]. Re-bleeding-free survival was defined as the period between variceal embolization and the date of re-bleeding or last follow-up visit. Laboratory tests were performed the day before and the day after treatment, and changes in serum concentrations of aspartate aminotransferase (AST), alanine aminotransferase (ALT), and total bilirubin (T-bil) were examined for the presence of Grade 3 adverse events using the Common Terminology Criteria for Adverse Events (CTCAE version 4.0). CECT was also performed after embolization.

### Procedure

The percutaneous transhepatic route was selected as the first-choice approach. However, the transileocolic route was chosen in cases where the portal vein was obstructed on preoperative imaging and for patients in whom the percutaneous transhepatic route had already been tried at another center and had encountered difficulty breaking through the portal vein obstruction. Patients treated by the transhepatic route underwent local anesthesia by injecting subcutaneous lidocaine, and the portal vein was punctured using the 21-G initial puncture needle included in the two-step puncture kit (MERIT MAK NV™; Medikit, Tokyo, Japan) under ultrasonographic and fluoroscopic guidance. After puncture, a 0.035-inch hydrophilic guidewire (TERUMO, Tokyo, Japan) and a 4- to 7-French sheath were inserted into the portal vein. In patients who were treated via the transileocolic route, laparotomy was performed under general anesthesia and the ileocolic vein was directly punctured with an 18-G indwelling needle, and a 0.035-inch hydrophilic guidewire and 7- to 10-French sheath was inserted. A 4- to 5.2-French catheter or balloon catheter (Royal Flush plus; Cook, Bloomington, IN, USA, or Medikit Angiography Catheter; Medikit, or Selecon MP catheter II; TERUMO) and 1.9- to 2.2-French microcatheter or micro-balloon catheter (Attendant SP, Attendant Nexus; TERUMO, or Masamune; Fuji Systems Corporation, Tokyo, Japan, or LOGOS GrandMASTER; Piolax Medical Devices, Yokohama, Japan, or Pinnacle Blue®27; Tokai Medical Products, Aichi, Japan) were used coaxially or in parallel. In all cases, 0.014- to 0.018-inch micro-guidewires were used.

Breaking through the portal vein stenosis or occlusion was attempted first as a treatment strategy. Variceal embolization was performed initially in all cases, regardless of whether the portal stenosis or occlusion was breached. This is because varices shrink once hepatopetal blood flow in the portal vein is restored, which can make the varices difficult to cannulate. Stenting was added after variceal embolization in all cases where the portal vein stenosis or occlusion was able to be penetrated. As embolic materials, coils, 5% ethanolamine oleate with iopamidol (EOI) containing gelatin sponge and N-butyl cyanoacrylate (NBCA) were used alone or in combination. After the 4th of a total of 14 sessions, embolization materials were standardized so that the distal portions of varices were embolized with 5% EOI and the proximal portions were embolized with coils. A self-expanding bare stent (SMART; Cordis, Miami, FL, USA, or Epic; Boston Scientific, MA, USA) with a diameter of 8, 10, or 12 mm (2–4 mm larger than the non-stenotic main portal vein) was used for stent placement, and portal vein balloon dilation (Mustang; Boston Scientific) was performed before and after stent placement. The coils used were C-stopper (Piolax Medical Devices), Tornado (Cook), Target XL (Stryker Corporation, Fremont, CA, USA), POD, POD packing, and RUBY soft (Penumbra, Alameda, CA, USA). The access route for the percutaneous transhepatic route was embolized using a mixture of contrast media and gelatin sponge. For the transileocolic route, the puncture site was ligated by the surgeon.

### Statistical analysis

Fisher’s exact test was used for categorical data. Kaplan–Meier analysis and the log-rank test were used to compare re-bleeding-free survival (stenting and embolization group versus embolization-only group). Statistical analyses were conducted using GraphPad Prism version 8 (GraphPad Software, San Diego, CA, USA). Values of *p* < 0.05 were considered statistically significant.

## Results

**Fig. 2:**
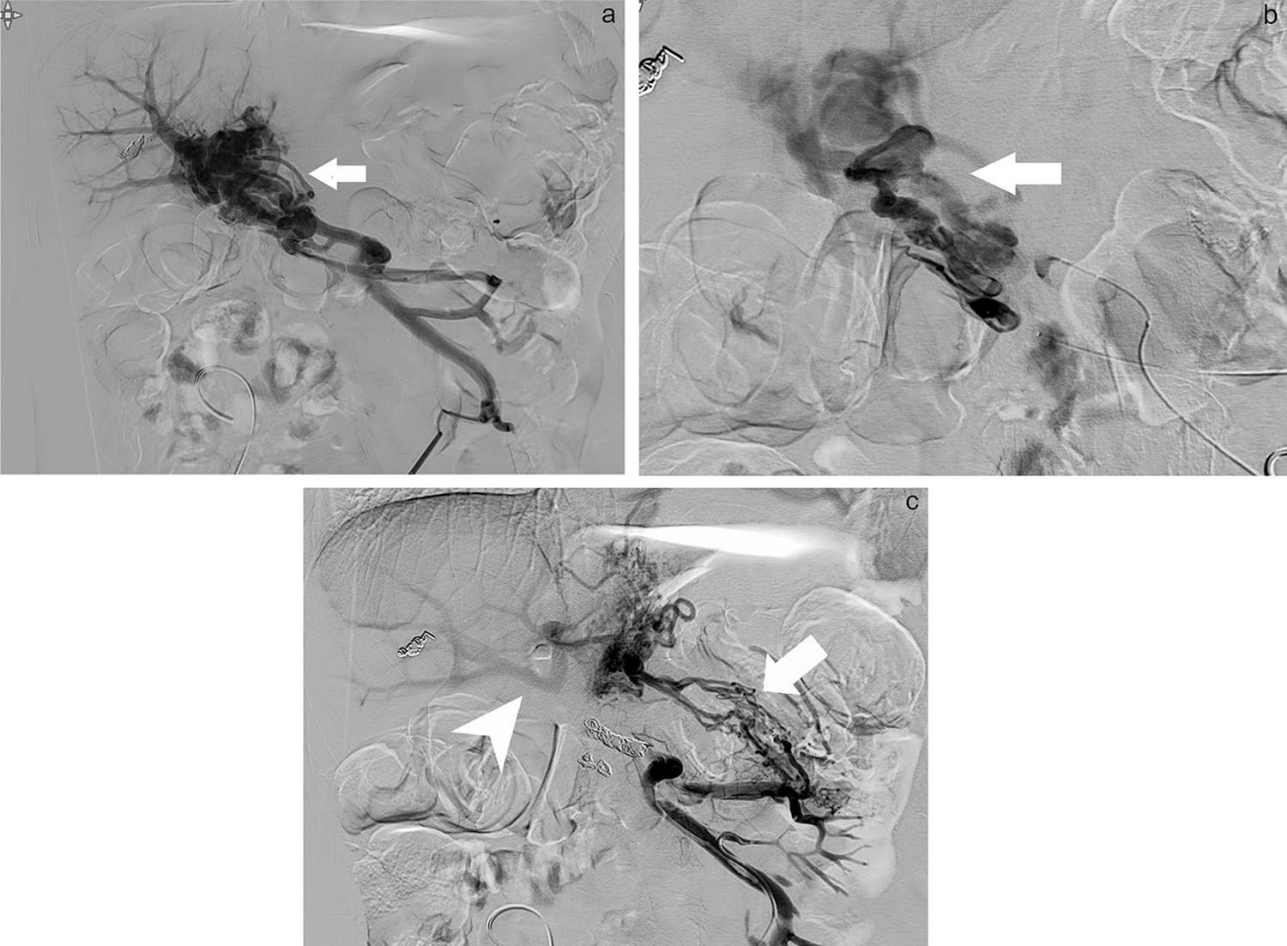
A 63-year-old man who had undergone pancreaticoduodenectomy and portal vein repair for pancreatic cancer underwent embolization of the hepatopetal portal venous varices 5 years after surgery (Case 10). The catheter was inserted from the ileocolic vein under a surgical approach. **a** Digital subtraction angiography (DSA) before embolization. Ectopic varices and intrahepatic portal vein are visualized. White arrow: ectopic jejunal varices. **b** Selective DSA from the first jejunal vein. Ectopic varices and intrahepatic portal vein are visualized. After this procedure, ectopic varices were embolized with metallic coils following a mixture of gelatin sponge and 5% ethanolamine oleate with iopamidol (EOI). White arrow: ectopic jejunal varices. **c** DSA after embolization from a superior mesenteric vein: Ectopic varices disappear, and a new collateral vein appears. White arrow: hepatopetal portal venous collaterals in the hepatogastric ligament. White arrowhead: intrahepatic portal vein

**Fig. 3:**
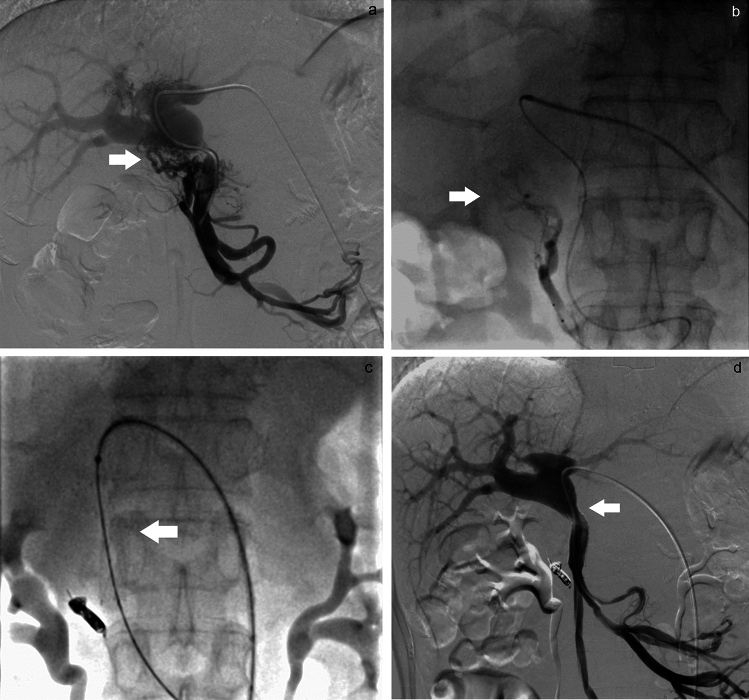
A 67-year-old man who had undergone subtotal stomach-preserving pancreaticoduodenectomy for pancreatic cancer underwent embolization of hepatopetal portal venous varices 4 years after surgery (Case 11). The catheter was inserted from the portal vein using a percutaneous transhepatic approach. **a** DSA before embolization and stent placement from a superior mesenteric vein. Ectopic varices and intrahepatic portal vein were visualized. White arrow: jejunal ectopic varices. **b** Embolization of ectopic jejunal varices. The distal portions of the varices were embolized with a mixture of gelatin sponge and 5% EOI under balloon occlusion. The proximal portions of these varices were then embolized with coils. White arrow: ectopic jejunal varices. **c** A metallic stent (Epic 12 mm × 4 cm) was placed over the stenotic region of the portal vein. White arrow: metallic stent. **d** DSA after embolization and stent placement from a superior mesenteric vein. Ectopic varices disappeared. White arrow: portal vein stenosis improved after portal vein stenting and balloon dilatation

**Fig. 4:**
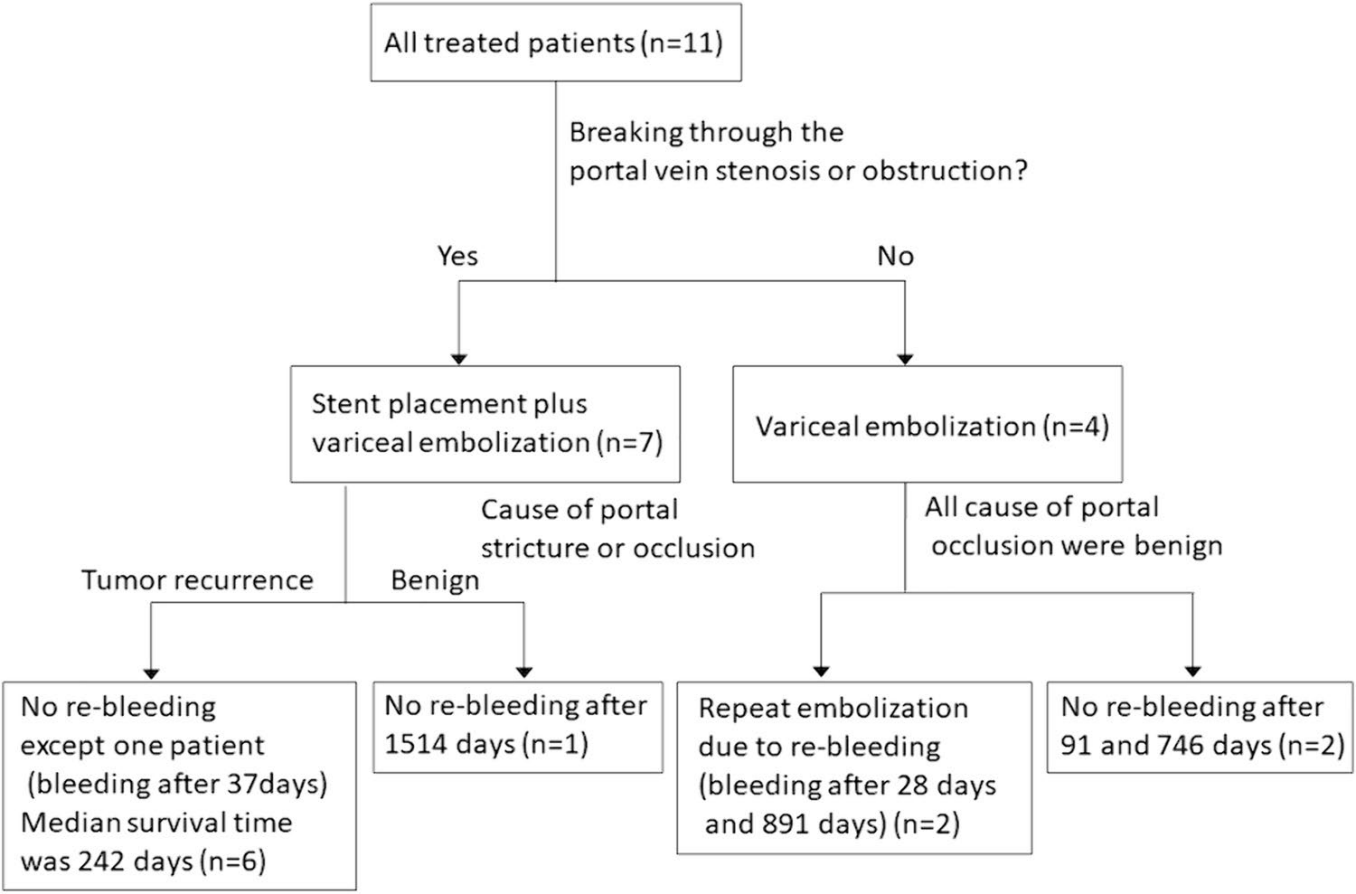
Breakdown of the treatment methods for bleeding ectopic jejunal varices in all 11 patients and the presence or absence of re-bleeding from ectopic jejunal varices

The clinical findings for all 11 patients are summarized in Table 1. The patients were divided into benign stenosis and tumor recurrence groups, and the clinical course of all sessions of treatment for each are shown in Tables 2 and 3. Four patients (7 sessions) underwent variceal embolization only (Fig. 2a–c), and the remaining seven patients (7 sessions) underwent portal vein stenting and variceal embolization (Fig. 3a–d). Ectopic variceal bleeding in the tumor recurrence group occurred at a mean of 25.0 months after surgery (range 11.4–46.4 months), and bleeding in the benign stenosis group occurred at a mean of 72.1 months after surgery (range, 2.4–240 months). Penetration of portal occlusion or stenosis was achieved significantly less frequently in the benign stenosis group (1 of 8 sessions) than in the tumor recurrence group (6 of 6 sessions; *p* = 0.0047). Percutaneous transhepatic route was used in 2 sessions and transmesenteric venous route was used in 12 sessions. As embolization materials, 5% EOI was used in 1 session, coils in 1 session, coils and 5% EOI with gelatin sponge and NBCA in 1 session, and coils and 5% EOI with gelatin sponge in all the remaining 11 sessions. Technical success was achieved in all 14 sessions (100%). Post-embolization angiography showed the presence of renewed hepatopetal collaterals other than the embolized varices in 6 of 14 sessions (Fig. 2c). In the follow-up period of all 14 sessions in 11 patients (range 28–2012 days; mean 602 days), 1 patient developed gastrointestinal bleeding due to varix recurrence within 1 month after initial treatment and required re-treatment. In all other sessions, no re-bleeding was observed within 1 month; clinical success rate was 92.9% (13 of 14 sessions). Two patients experienced recurrence of variceal bleeding more than 2 years after the endovascular treatment, at treatment intervals of 1900 days (Case 3) and 891 days (Case 4). The varices that caused this re-bleeding did not represent reopening of the initially treated route, but rather a new, different route. Figure 4 provides a flowchart breaking down treatment methods for bleeding ectopic jejunal varices in all 11 patients and the presence or absence of re-bleeding from ectopic jejunal varices. As for post-treatment complications, sepsis occurred in one session and resolved with antibiotic therapy. In four sessions, ascites appeared or increased, but all such cases were controllable with medical treatment. No post-treatment laboratory tests showed elevated AST, ALT, or T-bil levels (Grade 3 or higher in CTCAE version 4.0) that would suggest liver failure. No liver infarction was observed on CECT after embolization.

**Table 1:** Clinical findings at initial treatment in 11 patients with postoperative portal venous stenosis or occlusion

No	Age (years)	Sex	Primary disease	Surgical procedure	Time to bleeding after surgery (days)	Cause of portal stricture or occlusion (benign/tumor recurrence)
1	67	M	Pancreatic cancer	SSPPD	638	Tumor recurrence
2	74	M	Chronic pancreatitis	HJ	7300	Benign
3	57	M	Chronic pancreatitis	SSPPD	946	Benign
4	68	M	Gallbladder cancer	Right lobectomy + HJ	829	Benign
5	58	F	Bile duct cancer	SSPPD	559	Tumor recurrence
6	76	M	Pancreatic cancer	SSPPD	692	Tumor recurrence
7	68	M	Pancreatic cancer	SSPPD	347	Tumor recurrence
8	66	M	Pancreatic cancer	SSPPD	74	Benign
9	49	M	Pancreatic cancer	SSPPD + PVR	919	Tumor recurrence
10	63	M	Pancreatic cancer	PD + PVR	1814	Benign
11	67	M	Pancreatic cancer	SSPPD	1410	Tumor recurrence

*SSPPD* subtotal stomach-preserving pancreaticoduodenectomy, *HJ* hepaticojejunostomy, *PVR* portal vein repair, *PD* pancreaticoduodenectomy

**Table 2:** Clinical course in eight sessions of the benign stenosis group

No	Route	Treatment (embolic agents)	Technical success	Clinical success	Complications	Overall survival (days)	Period to retreatment (days)	Time to re-bleeding at the same site (days)	Status
2	TR	VE (EOI)	Success	Success	Sepsis	91	NA	None	Unknown
3–1	TR	VE (coil)	Success	Failure	None	NA	28	28	Alive
3–2	TR	VE (coil, EOI + GS, NBCA)	Success	Success	None	NA	1900	1900	
3–3	TR	VE (coil, EOI + GS)	Success	Success	None	2012	NA	None	
4–1	TR	VE (coil, EOI + GS)	Success	Success	Ascites	NA	891	595	Unknown
4–2	TR	VE (coil, EOI + GS)	Success	Success	None	994	NA	None	
8	PTR	SP + VE (coil, EOI + GS)	Success	Success	Ascites	1514	NA	None	Alive
10	TR	VE (coil, EOI + GS)	Success	Success	None	746	NA	None	Alive

*NA* not applicable, *TR* transileocolic route, *PTR* percutaneous transhepatic route, *SP* stent placement, *VE* variceal embolization, *NBCA* n-butyl-2-cyanoacrylate, *EOI* ethanolamine oleate iopamidol, *GS* gelatin sponge

**Table 3:** Clinical course in six sessions of the tumor recurrence group

No	Route	Treatment (embolic agents)	Technical success	Clinical success	Complications	Overall survival (days)	Period to retreatment (days)	Time to re-bleeding at same site (days)	Status
1	TR	SP + VE (coil, EOI + GS)	Success	Success	None	190	NA	None	Dead
5	TR	SP + VE (coil, EOI + GS)	Success	Success	Ascites	227	NA	None	Unknown
6	TR	SP + VE (coil, EOI + GS)	Success	Success	None	205	NA	None	Dead
7	TR	SP + VE (coil, EOI + GS)	Success	Success	Ascites	78	NA	None	Dead
9	TR	SP + VE (coil, EOI + GS)	Success	Success	None	319	NA	None	Dead
11	PTR	SP + VE (coil, EOI + GS)	Success	Success	None	242	NA	None	Dead

*NA* not applicable, *TR* transileocolic route, *PTR* percutaneous transhepatic route, *SP* stent placement, *VE* variceal embolization, *EOI* ethanolamine oleate iopamidol, *GS* gelatin sponge

**Fig. 5:**
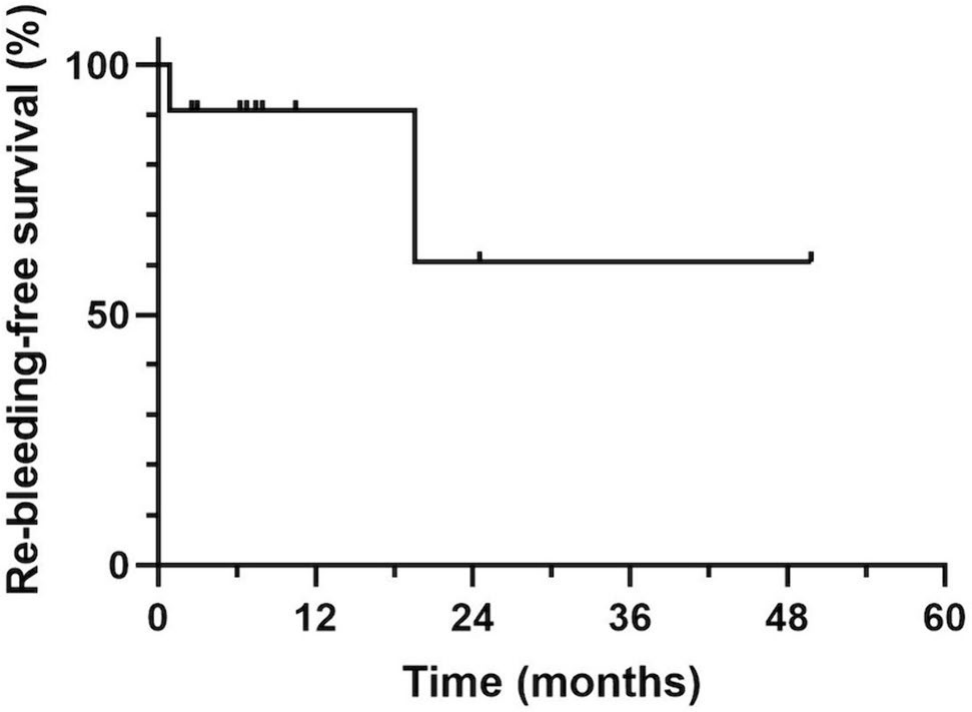
Kaplan–Meier curves displaying estimated 1- and 2-year re-bleeding-free survival rates after embolization of ectopic varices (90.9% and 60.6%, respectively)

**Fig. 6:**
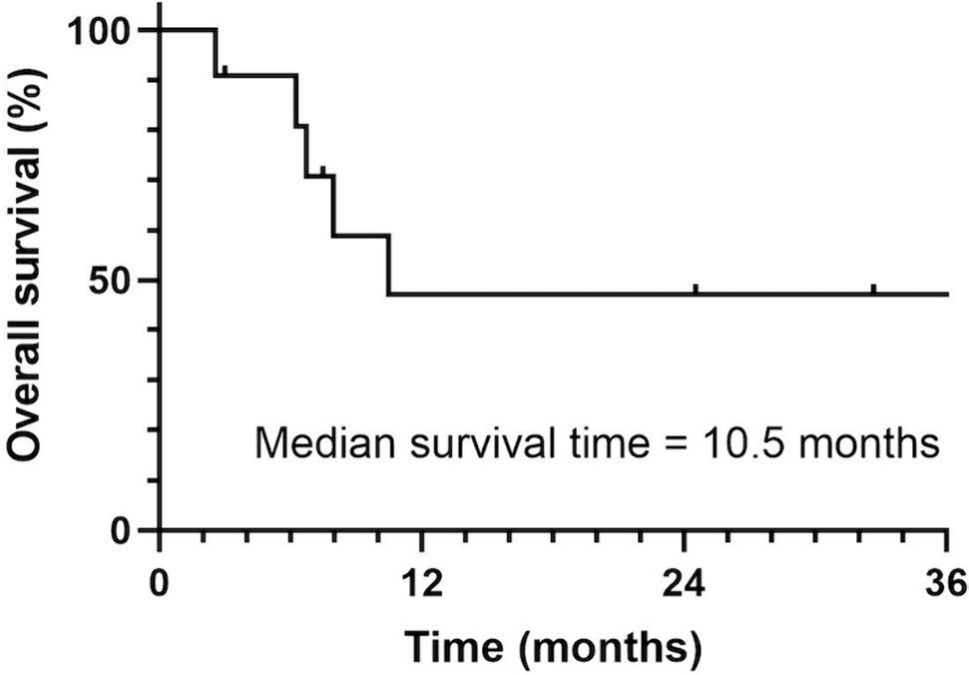
Survival curves. Kaplan–Meier curve displaying overall survival in all patients after embolization of ectopic varices. Median survival time is 10.5 months

Details of the stented patients are shown in Table 4. Of the seven patients who underwent stenting, four subsequently received continuous intravenous heparin infusion and were switched to oral antiplatelet therapy, two received both oral antiplatelet and anticoagulant therapy, and one received oral antiplatelet therapy alone. Six patients in the tumor recurrence group developed ectopic jejunal variceal bleeding during untreated follow-up or during chemotherapy and/or radiation therapy when tumor recurrence was detected. In all cases, chemotherapy was started or resumed as soon as possible after the treatment of varices. However, stent occlusion occurred in five of the seven patients, all in the recurrence group. The median time to stent occlusion in these five patients was 98 days (range 7–184 days). One patient developed bloody bowel discharge 37 days after treatment, but none of the other four patients showed bloody bowel discharge or progression of anemia during follow-up. As an additional treatment, one patient underwent additional portal vein stenting, but the stent showed reocclusion after one month. The remaining four patients did not receive additional endovascular treatment. One- and 2-year re-bleeding-free survival rates after first endovascular therapy in all 11 patients were 90.9 and 60.6%, respectively (Fig. 5). No significant difference in re-bleeding-free survival was identified between the stenting and embolization group and the embolization-only group (*p* = 0.13). Re-bleeding rates in the benign stenosis and tumor recurrence groups were 40% (2/5) and 0% (0/6), respectively. Median overall survival was 10.5 months (range 2.6–66 months) for all patients (Fig. 6), 32.7 months (range 2.9–66.0 months) for the benign stenosis group of five patients, and 7.1 months (range 2.6–10.5 months) for the tumor recurrence group of six patients. Five of the 11 patients died during follow-up, and all of them were in the tumor recurrence group.

**Table 4:** Clinical course after portal venous stent placement in seven patients

No	Cause of portal stricture or occlusion	Stent			Patency period (days)	Last stent status	Additional endovascular treatment
		Product name	Diameter (mm)	Length (cm)			
1	Tumor recurrence	SMART	8	6	98	Occlusion	No additional endovascular treatment
5	Tumor recurrence	SMART	10	6	163	Occlusion	Additional stent placement
6	Tumor recurrence	SMART	10	6	96	Patent	NA
7	Tumor recurrence	SMART	10	6	41	Occlusion	No additional endovascular treatment
8	Benign	SMART	8	6	1514	Patent	NA
9	Tumor recurrence	Epic	10	8	7	Occlusion	No additional endovascular treatment
11	Tumor recurrence	Epic	12	4	184	Occlusion	No additional endovascular treatment

*NA* not applicable

## Discussion

In this study, 1- and 2-year re-bleeding-free survival rates after the first endovascular therapy in all 11 patients were 90.9 and 60.6%, respectively. Even in patients who experienced re-bleeding, repeated treatment was successful. As a result, most patients treated with variceal embolization achieved long-term prevention of variceal bleeding. On the other hand, even in the absence of gastrointestinal bleeding, the formation of jejunal varices with portal vein occlusion or stenosis may be detected incidentally on CECT. Whether endovascular treatment should be performed for these non-bleeding ectopic varices remains controversial. No high-risk findings are currently known for these ectopic varices that could result in variceal bleeding, unlike the situation for gastroesophageal varices. Given the invasiveness of treatment, little justification may exist for treating non-bleeding cases to prevent future bleeding.

Stenting is an option for the endovascular treatment of ectopic varices in the hepatopetal collaterals formed by occlusion of the main portal vein. However, stenting may not be feasible, particularly in cases with long-term portal vein occlusion. In this study, the rate of portal vein breakthrough was significantly worse in the benign stenosis group than in the tumor recurrence group. Shim et al. performed portal stenting first, followed by embolization when varicose blood flow remained, but several authors have reported that cases with stenting alone re-bled immediately after treatment and underwent additional variceal embolization [8, 9]. As a treatment strategy, we performed variceal embolization first, followed by stenting, even in cases where the portal vein occlusion was penetrated and stenting was possible. The merit of performing embolization before stenting is that the varicose pathway is wide and easier to select with a catheter. The addition of variceal embolization after stenting has two advantages. First, embolization prevents recurrence in the same varices even if the stent becomes occluded. Second, embolization of the portal collateral vessels can be expected to increase hepatopetal blood flow in the stented main portal vein, which may lead to long-term stent patency. In this study, the mean patent duration of portal vein stenting in the malignant stenosis group was 3.3 months, which tended to be shorter than those reported by Kim et al. (mean 7.3 ± 7.7 months) and Yamakado et al. (mean 11.9 ± 12.9 months) [13, 14]. This result may be due to the different patient background in the present study, which included only patients with variceal bleeding and did not include cases of uncontrolled ascites or treatment of asymptomatic cases, and the smaller sample size compared to these previous studies.

In this study, blood transfusion every day to every 3rd day was required before all 14 sessions, but blood transfusion was no longer required after session in clinical success cases and the patients can be discharged from the hospital. One reason for the very high clinical success rate (92.9%) was that most sessions (85.7%) were treated via a transileocolic route. Variceal embolization was thus possible even when stenting via the transileocolic route was impossible. Some authors have reported no re-bleeding after variceal embolization during follow-up ranging from a few months to about 10 years [5–9, 11, 12]. During the follow-up period of this study, two patients in the benign stenosis group (Cases 3 and 4) experienced re-bleeding from ectopic jejunal varices and were again treated with embolization. In the tumor recurrence group, none of the six patients experienced variceal re-bleeding. This might seem to indicate that the benign stenosis group performed worse than the tumor recurrence group in terms of prevention of re-bleeding. However, based on the fact that two of these sessions (3–3, 4–2) took place more than 2 years after the previous treatment, we inferred that the benign stenosis group had a background of long-term viability, resulting in a higher re-bleeding rate. To our knowledge, no previous reports have described re-embolization with varix recurrence several years after embolization. This and the previous studies suggest that embolization of ectopic varices in hepatopetal portal collaterals due to extrahepatic portal vein occlusion or stenosis is effective for long-term hemostasis, offers the advantage of repeatable treatment, and can be expected to prolong overall survival for several years by preventing death due to variceal bleeding, particularly for benign stenosis.

It has been speculated that a risk of liver failure and hepatic encephalopathy may arise due to reduced intrahepatic portal blood flow in cases of variceal embolization alone [15]. In this study, post-embolization angiography showed the presence of renewed hepatopetal collaterals other than the embolized varices in 6 of 14 sessions. Moreover, no patients developed liver failure or hepatic encephalopathy, and no hepatic infarction was observed on post-treatment CECT. Several authors have reported no cases of liver failure immediately after embolization [5, 7–9, 11, 12]. Taken together, such findings suggest the presence of a third hepatopetal portal venous pathway other than the varices pathway and main portal vein, and intrahepatic portal venous blood flow may have been maintained within acceptable limits after embolization.

Several reports have described stent patency rates of 60–100% with months to years of follow-up in studies of stenting for portal vein stenosis [13, 14, 16, 17]. Kim et al. reported that the stent patency period was significantly shorter with malignant stenosis than with benign stenosis (patency period: 30.1 ± 25.6 months in the benign group and 7.3 ± 7.7 months in the malignant group) [13]. Ohgi et al. reported median stent patency of 69.9 months (range 14.5–103.9 months) in six patients with stenting for benign portal vein stenosis, achieving long-term stent patency [18]. Although whether variceal embolization is necessary at the time of portal vein stenting remains controversial, these previous reports suggest that variceal embolization is recommended to prevent re-bleeding, particularly in cases of malignant stenosis, due to the risk of variceal recurrence from early stent occlusion after treatment. One patient in this study who experienced early stent occlusion 7 days after portal stenting and variceal embolization had no anemia or bloody bowel discharge. This suggests that variceal embolization is effective.

Our study showed several limitations. First, ectopic jejunal varices in the hepatopetal portal collaterals due to extrahepatic portal vein occlusion or stenosis are rare, so the number of accumulated cases remains small and prospective studies are difficult. As the numbers of cases at a single institution will be limited, cases may need to be accumulated from multiple institutions to allow valid statistical investigation of the efficacy and safety of treatment effects. Second, treatment methods, devices, and embolic materials were very variable in this study. This situation may represent a key limitation for discussing the results in terms of the treatment technique in this comparatively limited number of subjects.

We concluded that embolization of ectopic jejunal varices in the hepatopetal portal collaterals due to extrahepatic portal vein occlusion or stenosis can be considered a safe and effective therapy for long-term hemostasis of uncontrollable bleeding. Furthermore, this procedure can be repeated in cases of re-bleeding.
